# Self or nonself: end of a dogma?

**DOI:** 10.3389/fimmu.2025.1595764

**Published:** 2025-05-08

**Authors:** Marie Duhamel, Michel Salzet

**Affiliations:** ^1^ Univ. Lille, Inserm, Centre Hospitalier Universitaire (CHU) Lille, U1192 - Protéomique Réponse Inflammatoire Spectrométrie de Masse (PRISM), Lille, France; ^2^ Institut Universitaire de France, Ministère de l’Enseignement supérieur, de la Recherche et de l’Innovation, 1 rue Descartes, Paris, France

**Keywords:** self-non-self, autoimmunity, microchimerism, hygienist theory, molecular mimicry hypothesis, microbiota, trained innate immunity

## Abstract

Immunologists generally view the notion of self and non-self as part of a broader, more contextual understanding of immune function, rather than a rigid dogma. While the classical paradigm that the primary role of the immune system is to recognize and eliminate anything foreign once provided a unifying basis for explaining tolerance and rejection, numerous discoveries have focused attention on how immune responses are finely tuned by a range of contextual cues, including tissue signals, hygienist theory, molecular mimicry, symbiotic microbes, metabolic factors and epigenetic modifications. Maternal-fetal tolerance and the persistence of microchimeric cells in adults demonstrate that genetically foreign cells can be actively integrated into the host, challenging the simple assumption that ‘foreign’ equals unconditional attack. Similarly, research into the microbiome, the virome and the phenomenon of trained innate immunity has shown that there can be beneficial or even essential relationships between the body and what has traditionally been labelled ‘non-self’. Over the last decade, the idea that the immune system strictly enforces a binary distinction has instead evolved towards a model in which it continuously interprets signals of damage or perturbation, manages complex ecological relationships with commensal or latent organisms, and recalibrates according to the organism’s life stage and environment. There remains a recognition that clonal deletion and negative selection in the thymus, together with MHC-bound peptide recognition, still underlie many core processes, and in certain clinical contexts, such as acute transplant rejection or the prevention of autoimmunity, an approximate self-non-self-categorization is directly relevant. Overall, however, the field recognizes that ‘self’ is not a static attribute defined once and for all, but rather a dynamic and context-dependent state that continues to be shaped by microbial symbioses, epigenetic reprogramming and immunoregulatory networks throughout an individual’s lifespan.

## Historical foundations before mid-century

The question of what constitutes the ‘self’ and how living organisms maintain their integrity against external threats has preoccupied thinkers from diverse fields, including philosophy, biology and medicine, for centuries. However, it was not until the late nineteenth and early twentieth centuries that these questions found a consolidated theoretical anchor in immunology. In the classical view that emerged, the basic function of the immune system was to identify, label as non-self, and eliminate all that is foreign, while preserving all that is self. This way of thinking, formalized early on by pioneers such as Paul Ehrlich (1), Elie Metchnikoff (2, 3), and Jules Bordet (4, 5), gained momentum during in the first half of the twentieth century. To understand why the self-non-self-dogma became such a cornerstone, one must first appreciate the social and scientific climate that gave rise to it in the decades surrounding the early twentieth century. Although modern immunology is often said to have begun with Louis Pasteur and Robert Koch’s focus on infectious disease, its conceptual roots lie in a mixture of philosophical speculation, empirical observation and clinical necessity. Early philosophical concepts of identity, such as those articulated by John Locke, explored the nature of the self in purely cognitive or spiritual terms. John Locke’s (6) work on personal identity in An Essay *Concerning Human Understanding (1690)* explored what it meant to be ‘oneself’, emphasizing consciousness and memory rather than mere bodily continuity. He wondered whether personal identity might lie in memory and consciousness rather than in a stable, unchanging material substrate. Although Locke was not directly concerned with immune function, his exploration of identity highlights a fundamental tension that would echo through immunological discourse centuries later: Is the self an absolute, fixed entity, or is it to be understood as a dynamic construct shaped by processes, interactions and contexts? Advances in experimental medicine gradually reframed this philosophical question in biological terms. Locke did not speak of ‘non-self’ in an immunological sense, but he laid the philosophical foundations for later debates about identity, including the biological basis of selfhood. It was only with the advent of vaccinology and microbiology that the concept of ‘self’ began to take on a more biological dimension.

The era of Edward Jenner, (7) who introduced vaccination against smallpox in 1796, marked a shift from vague notions of bodily self-protection to concrete evidence that the body could be primed to resist specific pathogens. This specificity implied that there must be a mechanism capable of distinguishing between different external challenges. Observers marveled that a mild exposure could result in robust, long-lasting protection from a far more virulent challenge. This phenomenon implied that the body could develop a memory of what it had encountered and react more efficiently upon re-encounter. Soon after, Louis Pasteur (8) and Robert Koch (9) aid the foundations of germ theory, showing that microbes were often the cause of infectious diseases and that the organism needed a strong internal defense. Robert Koch’s postulates changed the way scientists thought about microbes. For the first time, diseases such as anthrax, tuberculosis and cholera were definitively linked to specific pathogens, each recognized as an external biological entity capable of invading the body. Observers naturally asked how the body could detect and repel such invaders. But the question of how the immune system could recognize pathogens with such precision remained unanswered. Metchnikoff, often hailed as the father of cellular immunology, discovered phagocytes and proposed the idea that certain host cells were engaged in active surveillance against foreign entities (3). Elie Metchnikoff’s observation of phagocytosis in starfish larvae and subsequently in mammalian cells, underscored an elementary capacity for cellular recognition and engulfment of foreign bodies, but also raised the question of how these innate responses might deal with subtle or partial forms of foreignness (3). He did not use the term ‘non-self’ systematically, but his demonstration of phagocytic cells engulfing and destroying foreign material provided an early framework for how cells might distinguish the body’s own structures from external invaders. But where was the boundary between threatened ‘self’ and invading ‘non-self’?

In the early 1900s, Jules T. Portier and Charles Richet studied phenomena of immune hypersensitivity and eventually coined the term “anaphylaxis”. Their studies showed that the immune response could be pathologically misdirected, suggesting that “foreign” triggers sometimes caused excessive and damaging reactions (10). Although not explicitly called self/non-self-discrimination, their work underlined how recognition events can have paradoxical effects on health and disease. Paul Ehrlich furthered these investigations when he formulated his side-chain theory and introduced the concept of horror autotoxicus, insisting that an immune system that attacked its own tissues would be fatally self-destructive (11). This assumption that the healthy organism avoids immune reactivity against itself and reserves aggression for foreign invaders took on the force of dogma. Although he did not explicitly say ‘non-self’, his view that the organism must somehow be protected from its own destructive immunological reactions helped to shape the notion of self-tolerance. Ehrlich meticulously showed that the body produced specific antibodies against toxins or pathogens but seemed to avoid producing equally potent responses against its own tissues. This was not just a curiosity, but a vital necessity, since any large-scale immune attack on the self was likely to be fatal. By implication, anything outside this tolerant boundary would be recognized as foreign and attacked. But cracks began to appear in this early conceptualization. Even Ehrlich was aware that the boundary between self and non-self was vulnerable to breakdown, leading to pathologies that would later be called autoimmune diseases. While this was an elegant conceptual leap, Ehrlich’s theory was largely silent on the deeper question of how the self-came to be recognized as ‘safe’. Nevertheless, it provided strong evidence that the immune system could distinguish self from foreign under normal physiological conditions. As the twentieth century began, Jules Bordet (5), Karl Landsteiner (12), and others expanded on humoral immunity, showing that serum components (later understood to be immunoglobulins (13, 14) and complement factors (15) recognized specific chemical structures. The specificity was astonishing: immune sera could distinguish between subtle differences in chemical or biological molecules. Researchers were understandably amazed by the adaptive capacity of the immune response, but the phenomenon of self-tolerance was less often discussed in this context. Instead, the focus was on how to enhance or induce responsiveness to pathogens. Nevertheless, small cracks began to appear in the self-non-self-narrative. Clinical reports described conditions where the immune system attacked the body, such as hemolytic anemia (16) or rheumatoid arthritis (17), suggesting that the line between self and nonself was not infallible. But such observations were interpreted mainly as pathologies, malfunctions of a system presumed fundamentally geared to preserve self and eliminate foreign antigens. This framing helped maintain the primacy of the dogma: pathological exceptions did not necessarily undermine the broader theoretical stance.

In the 1930s and 1940s, interest in transplantation, initially driven by the desire to repair war injuries, brought new data. Even as these ideas took hold, competing and complementary observations began to accumulate. Joseph (Jules) Hamburger (18, 19), Jean Dausset (20, 21), and Barry D. Kahan (22) advanced transplantation immunology by demonstrating that the success or failure of organ transplants was intricately linked to the matching of specific genetic loci (HLA in humans). The discovery of major histocompatibility complexes (MHC) and the demonstration of how T cells recognize processed peptides in the context of MHC molecules lent further credence to the idea that ‘self-markers’ underpin immune recognition. Attempts to transplant tissues from one individual to another often failed, leading surgeons and scientists to question what triggered the rejection of these tissues that might have been beneficial from a purely functional standpoint. The impetus to overcome graft rejection laid the groundwork for research into the genetic barriers to transplantation. Meanwhile, the conceptual leap that linked rejection to the principle of self-non-self was about to become more explicit.

In short, the period before mid-century was characterized by a gradual convergence of ideas: from philosophical musings on identity, to the demonstration that the body “remembers” and fights specific pathogens, to the identification of molecular complexities in serum and cells that confer selective reactivity. But the formal articulation of self-non-self-discrimination as a guiding principle was still in flux. It would coalesce and become a dogma primarily through the work of a new generation of immunologists operating in the post-1950 landscape. Before proceeding, it is important to note that this early period, although overshadowed by the striking successes of vaccination and infectious disease control, served as the foundation upon which modern immunological theory was built. Researchers cultivated the notion that the immune system was inexorably oriented toward preserving the integrity of the host while eliminating perceived threats. The self-non-self-dogma was thus ready to crystallize once someone provided a unifying, mechanistic model. That person was Frank Macfarlane Burnet, whose theory of clonal selection would become the lynchpin of mid-century immunology (23).

## Mid-century codification of the self–nonself paradigm

The post-1950 era in immunology was marked by a drive to synthesize disparate observations into coherent models. This was the time when immunology solidified as a formal discipline, with journals, societies, and conceptual frameworks that would dominate for decades. In this context, Frank Macfarlane Burnet emerged as one of the leading voices. His theory of clonal selection, first articulated in the late 1950s and expanded in subsequent publications, argued that each lymphocyte clone carries a unique antigen receptor (23). Upon encountering its specific antigen, the clone is activated to proliferate and mount an immune response. Crucially, any clones recognizing self-antigens should be deleted or inactivated, thereby preventing autoimmunity and establishing tolerance. Burnet’s formulation was revolutionary in its explanatory power. It elegantly accounted for immunological specificity (each clone recognizes one antigen), memory (proliferation of that clone after encounter), and tolerance (negative selection of self-reactive clones). For many immunologists, it resolved fundamental mysteries that had been simmering beneath the surface. The concept of “forbidden clones” to be culled or suppressed fit well with the growing awareness that autoimmune processes were pathological aberrations. This model thus provided intellectual scaffolding for the notion that “normal” immunity was based on recognizing foreign antigens while ignoring the self. At the same time, Niels Jerne proposed the idea of an idiotypic network, suggesting that antibodies and T cell receptors regulate each other through interlocking frameworks of recognition (24, 25). Although Jerne’s focus was on the regulatory interplay of receptors rather than self-non-self-discrimination per se, his work complemented Burnet’s model by illustrating how the immune system might maintain homeostasis. If each receptor could be regulated by another, the system contained feedback loops that could keep self-reactivity in check. The underlying assumption remained that the system was fundamentally designed to distinguish the host’s own structures from anything foreign. The triumph of clonal selection theory also coincided with major breakthroughs in transplantation biology (19, 26, 27). Researchers unraveled the genetic basis of graft rejection. Dausset’s discovery of the HLA complex made it clear that recognition of transplanted tissues depended on major histocompatibility antigens (20, 21). The intricate polymorphisms of these molecules explained why graft acceptance was more likely in genetically matched individuals. These findings strengthened the self-non-self-dogma by identifying the “self-markers” that the immune system used as a baseline for recognition. The synergy between clonal selection theory and transplantation immunology led to the idea that alloreactive T cells simply responded to non-self MHC molecules. If the transplanted tissue carried MHC alleles that differed from the host, immune attack would be the natural outcome, consistent with the central precept that foreign cells induce immunity. Several lines of investigation across species supported this perspective, as histocompatibility complexes were discovered in mice (H-2) and other model organisms (28, 29). Researchers such as Jan Klein expanded the characterization of MHC genetics, revealing the extensive polymorphisms that shape immune responses (30). Such findings were widely interpreted as further validation of the dogma. During the 1960s and 1970s, immunological research flourished. Conceptual anchors included: (1) clonal selection as the generator of specificity and memory (31), (2) MHC molecules as key self-markers (32), (3) T-cell clonal expansion and the T-cell receptor gene 33, (4) the identity of foreign antigens as the prime mover in initiating immune responses, and (5) the assumption that tolerance to self is achieved mainly by eliminating or inactivating self-reactive clones (33). This dogma pervaded immunology textbooks and informed clinical interventions, such as early attempts at immunosuppression to prevent transplant rejection. The thought leaders of this era, Frank Burnet, Niels Jerne, Peter Medawar (34), and their disciples, set the intellectual tone. Nevertheless, even at the height of this conceptual hegemony, anomalies persisted. Phenomena such as immune privilege in certain tissues (e.g., eye, brain, and testes) and pregnancy tolerance (mother tolerating semi-allogeneic fetus) were not fully explained by the clonal selection narrative, at least not without adding further layers of complexity (35). Rare but well-documented cases of chimerism and naturally occurring blood cell micro-transfusions also defied the neat boundary implied by “foreign equals aggression” (36). Moreover, autoimmune diseases such as systemic lupus erythematosus (37), type 1 diabetes (38), and rheumatoid arthritis (39) continued to be reported, implying that mechanisms of tolerance to self were more fragile and complex than a simple “weeding out” of forbidden clones could explain. Yet these counterexamples were often treated as exotic exceptions. For the bulk of immunology, the self-non-self-perspective was robust and served as a practical framework for both basic research and therapeutic innovation.

In summary, the period from 1950 to roughly the early 1980s represents the classic apogee of the self-non-self-dogma. It was an era in which the excitement of theoretical coherence, the synergy with genetic and transplantation data, and the support of immunological experimentation at multiple levels converged. Burnet’s theory of clonal selection not only described immunology; it shaped how researchers approached questions of tolerance, autoimmunity, and vaccine design. The continued success of these models, despite the presence of some unresolved mysteries, created confidence that immunology had arrived at a unifying theory that could stand the test of time. Only in the following decades would the unexpected challenges posed by innate immunity, danger signals, microbial symbiosis, and systems biology begin to destabilize this once unquestioned dogma.

## Expanding and challenging the paradigm

With the classical version of self-non-self firmly in place by the early 1980s, immunology seemed to be on solid ground. But science rarely remains static. As the field expanded, a host of new data and concepts emerged, some reinforcing the established dogma, others undermining it. The first major conceptual shift came from deeper investigations of innate immunity. Until the 1980s, innate immunity was often relegated to simple, non-specific defense. The real intellectual energy surrounded adaptive immunity, B cells, T cells, immunological memory, precisely because the self-non-self-model saw the crux of discrimination in the adaptive repertoire. Charles Janeway challenged this emphasis in the late 1980s, proposing the concept of the “infectious non-self” (40). He argued that the immune system was fundamentally keyed to detect pathogen-associated molecular patterns (PAMPs), molecular motifs conserved among microbes but absent in the host. This perspective moved the focal point of discrimination from random foreign antigens recognized by lymphocytes to evolutionarily ancient pattern recognition receptors on innate cells. While Janeway’s vision still hinged on distinguishing self from microbial non-self, it shifted attention to the innate branch of immunity as an active, sophisticated participant in discrimination. Yet a more radical challenge emerged in the 1990s when Polly Matzinger introduced the “danger model.” Matzinger proposed that immune responses are triggered not by foreignness alone but by signals of damage or danger, such as those released by necrotic or stressed cells. In her model, context is everything (41). A self-antigen presented under non-danger conditions might be tolerated, whereas the same antigen released in the presence of tissue injury or inflammatory cues might provoke a strong immune response. The danger model decentered the self–nonself dichotomy, suggesting that “foreignness” is merely one among many cues. Critically, the model explained why the immune system sometimes responds to uninfected dying host cells (as in autoimmunity) or tolerates certain foreign antigens when they appear in a non-inflammatory context. Although the danger model was not universally accepted in all its specifics, it undeniably cracked the monolithic status of the self–nonself framework. Parallel to these theoretical developments, the field of transplantation immunology was undergoing profound changes. While earlier efforts had focused on matching donor and recipient HLA and using immunosuppressive drugs to dampen the adaptive response, researchers like Barry D. Kahan were demonstrating that T cell targeting with agents such as cyclosporine or tacrolimus could prolong graft survival dramatically (22). However, complete immunosuppression led to opportunistic infections and other complications, suggesting that a more nuanced approach was needed. Innovative immunologic strategies emerged to achieve “engraftment tolerance,” a state in which the recipient’s immune system coexists indefinitely with the transplanted organ without ongoing immunosuppression. Studies of microchimerism, in which a subset of donor cells persist in the recipient, revealed how the boundary between self and foreign could be strategically blurred to promote acceptance (42, 43). his phenomenon had echoes of fetal-maternal tolerance, which in turn challenged the notion that foreign tissues inevitably provoke rejection.

By the early 2000s, a surge of interest in innate immunity brought forth new revelations. Jules Hoffmann’s and colleagues work on the Toll pathway in Drosophila (44, 45), later linked to Toll-like receptors in mammals (46), demonstrated that innate immune responses are orchestrated by sophisticated genetic and signaling networks that sense microbial and even endogenous danger signals (47–49). This reinforced Janeway’s concept of PAMP recognition (50) but also introduced the recognition of damage-associated molecular patterns (DAMPs), in line with Matzinger’s danger model (41). The interplay of these innate signaling pathways eventually gave rise to the concept of “trained immunity,” championed by Mihai Netea and colleagues (51). They showed that innate cells, long thought to lack memory, could in fact develop enhanced or altered responsiveness to certain stimuli, thanks to epigenetic reprogramming. Trained immunity implied that host tissues might blur the lines between responding to foreign patterns and re-responding to subsequent endogenous or exogenous cues. At the same time, philosophical critiques by Alfred Tauber (52) and Thomas Pradeu (53) kept drawing attention to the complexities of immune identity. Tauber argued that immunology should be understood as a science of “negative knowledge,” concerned primarily with what is to be tolerated rather than what is to be destroyed. Pradeu introduced the idea of a continuous immune self, constantly monitored and shaped by interactions with microbes, tissues, and even transplanted cells (53). According to this perspective, the immune system is less a policeman patrolling a hard border than a manager of ongoing negotiations between host biology and environmental influences. The classical notion that “self is recognized by default” and “non-self is attacked” no longer seemed adequate to describe the multiplicity of states in which foreign elements might be tolerated (commensal bacteria, fetal cells, integrated viruses) or in which self-components might provoke aggression (autoimmunity, tumor immunity). The modern understanding of self and non-self in immunology does not derive from a single seminal finding, but rather from a broad accumulation of discoveries that illustrate the remarkable plasticity of the immune system. An instructive example comes from studies of fetal-maternal tolerance. In a paper published by Bianch (42), researchers reported that male fetal cells could persist in maternal blood for decades after pregnancy, revealing that the immune system often tolerates genetically foreign cells long-term. More recent investigations, such as those discussed by Moffett and Colucci (54), have further shown that maternal tolerance involves sophisticated regulatory networks in which specialized subsets of immune cells, including uterine natural killer cells and regulatory T cells, actively promote the survival of fetal tissues that bear paternally derived antigens. This capacity to embrace partial foreignness runs contrary to a strict binary model that would predict swift rejection of any non-self-antigen. A second line of evidence arises from research on innate immune memory, sometimes known as trained immunity. In their landmark paper Netea, Quintin, and van der Meer (55) described how monocytes and macrophages can undergo epigenetic remodeling after an initial encounter with microbial or inflammatory stimuli, thereby responding more robustly to subsequent challenges. This finding challenged the longstanding assumption that only lymphocytes (T and B cells) are responsible for immunological memory. It also showed that immune reactivity does not hinge solely on recognizing “foreign” antigens but can instead be modulated by prior inflammatory context, even if the triggering agent in a subsequent infection is antigenically distinct. Similar insights have emerged from the burgeoning field of microbiome research, exemplified by work from Honda and Littman (56). They documented how gut-resident commensal bacteria contribute to the development of key immune cell populations, such as regulatory T cells that help preserve intestinal homeostasis. Such findings imply that the immune system not only tolerates but also depends on certain microbial partners for normal maturation, making the presence of foreign genetic material an integral component of healthy immune function. In this context, it is also important to integrate the traditional hygienist theory or hygiene hypothesis, originally proposed by Strachan (57) (57), posited that limited exposure to infectious agents in early childhood might predispose individuals to hypersensitive allergic responses due to an imbalance between Th1 and Th2 immune responses (58). Initially controversial because of its simplicity and limited empirical basis, subsequent research has considerably expanded and nuanced this theory, highlighting its relevance across diverse immunological contexts (59, 60). Modern interpretations emphasize microbial diversity and continuous microbial exposure as critical for developing and regulating immune mechanisms. The shift from a simplistic Th1/Th2 dichotomy to a broader perspective involving regulatory T cells (Tregs), interleukin-10, and transforming growth factor-beta (TGF-β) underscores how microbial interactions induce immune tolerance (56, 61). Gut microbiota, particularly bacteria like Bacteroides fragilis and Clostridium species, significantly influence Treg differentiation, thereby protecting against autoimmune and inflammatory disorders (56) This realization problematizes any attempt to define nonself categorically as an immediate target for immune clearance. Meanwhile, the discovery of the human microbiome and virome fundamentally altered perceptions of biological individuality. If the body is host to trillions of microbes, many essential for normal physiology, does the immune system categorize them as foreign? Are they included in an extended sense of self? These questions became pressing when research showed that microbiota not only shaped immune development but also influenced neurological function, metabolic processes, and susceptibility to diseases ranging from inflammatory bowel disease to certain autoimmune conditions. In fact, molecular mimicry consisting in a phenomenon in which a pathogen expresses antigens that closely resemble the host’s own proteins (62, 63). Such similarity allows an immune response against the microbe to cross-react with self-antigens, meaning that antibodies or T cells raised to eliminate the pathogen can also bind host tissues, potentially triggering autoimmune disease (62, 63). This mechanism effectively blurs the line between “self” and “non-self”, a cornerstone of classical immunology, by breaking immune tolerance and causing the immune system to mistake self for foreign. For example, in rheumatic fever, antibodies produced against the Group A Streptococcus (*Streptococcus pyogenes*) M protein also recognize proteins in the heart (such as myosin and other cardiac tissue antigens), leading to inflammation and damage of heart valves (64). Likewise, infection with *Campylobacter jejuni* can precipitate Guillain–Barré syndrome through molecular mimicry: antibodies targeting the bacterium’s surface lipooligosaccharides cross-react with peripheral nerve gangliosides, causing demyelination of nerves and acute neuropathy (65). These examples illustrate how a normal protective immune response can inadvertently become autoimmune when confronted with a mimic, challenging the classical self/non-self-model and highlighting the importance of immune tolerance safeguards. In essence, molecular mimicry shows that infections can undermine self-tolerance and trigger autoimmunity, refining our understanding of immune recognition and the fine balance that normally protects against attacking one’s own tissues. Similarly, endogenous retroviruses and persistent viral infections further complicated the picture, revealing that viral genes and particles often integrated stably into host tissues, sometimes conferring evolutionary advantages (66, 67). Taken together this highlights that the immune response to persistent or latent viruses can shift from aggressive control to a regulated equilibrium, contingent on host genetics, microbiota interactions, and environmental factors and demonstrate that immune tolerance to aspects of the virome is not purely a passive event; rather, it involves dynamic immunoregulatory pathways that discern whether particular viruses pose a threat or can exist as asymptomatic, and in some cases beneficial, components of the host’s ecological network. Additional research continues to demonstrate that commensal viruses, much like commensal bacteria, can shape local and systemic immune responses, underscoring that viral “non-self” does not invariably trigger a classical rejection mechanism (68). These examples support a view that the immune response depends less on the mere fact of foreignness than on context, epigenetic changes, developmental cues, and ongoing ecological interactions. The notion of “self” thus emerges, not as a static property conferred by an individual’s genome alone, but as an evolving construct continually shaped by the presence of fetal cells in adult tissues, by the regulatory impact of commensal microbes, and by reprogramming events in innate immune cells. Each of these phenomena directly underscores the idea that the boundary separating self and non-self is neither absolute nor permanent but is dynamically regulated according to the biological circumstances in which the immune system finds itself. Moreover, an important element to be considered is the dynamic in course of ageing of the self-recognition. In fact, From the earliest stages of fetal development, the immune system is shaped by maternal inputs, including antibodies, cytokines, and, in some cases, transplacental cell trafficking that can lead to microchimerism (42). Microchimerism’s impact on immune responses becomes most apparent when one considers its links to autoimmunity (69) and potentially to tumor surveillance or progression (70). Specifically, microchimerism may offer a unique window on immunosurveillance by continuously stimulating the immune system at a low, non-pathogenic level. The persistent presence of genetically distinct microchimeric cells presents minor histocompatibility antigens or polymorphic markers that subtly challenge immune surveillance systems. This continuous low-level stimulation can maintain immune vigilance, potentially enhancing the detection of “altered self” targets, such as tumor cells, by maintaining immune cells in a heightened state of readiness without triggering chronic inflammation or autoimmunity (43). Evidence for this idea remains under active investigation, but some researchers theorize that microchimeric cells might act analogously to a low-level graft, consistently provoking mild stimulation of certain immune populations. On the other hand, if host microchimeric coexistence leads to chronic low-grade inflammation or a perpetual regulatory stance, the balance could shift unfavorably in contexts of incipient malignancy. A chronically inflamed environment, for instance, is known to support tumor progression through mechanisms like angiogenesis and immunosuppressive feedback loops. Chronic regulatory T cell expansion, necessary for tolerating genetically foreign cells, might also dampen tumor-specific cytotoxic responses. However, microchimerism remains controversial due to its potential link to pathological conditions. Chronic immune activation induced by microchimeric cells may predispose individuals to autoimmune diseases such as systemic sclerosis and autoimmune thyroiditis, highlighting the delicate balance in immune modulation (69). Emerging research further explores microchimerism’s role in tissue repair and regenerative medicine, suggesting involvement in tissue regeneration by modulating local inflammation and aiding repair processes (43, 71). Yet whether microchimerism confers a net protective effect (heightened vigilance via recognition of minor antigens) or a net permissive environment (tolerance or inflammation-induced immunosuppression) is likely to depend on the tissue in question, the individual’s genetic makeup, and the broader immunological context. Thus, for scientists studying immunosurveillance, microchimerism poses both a potential immunological asset, broader recognition capability and a liability if the tolerance mechanisms it fosters become hijacked by emerging tumor cells (71). Whereby maintaining heightened readiness for transformation events in host tissues. Thus, during this prenatal window, self–nonself distinctions are not rigidly fixed but emerge gradually, as the maturing immune system encounters paternal antigens in a controlled uterine environment and builds early tolerogenic networks (72). At birth, neonatal immunity remains highly plastic, depending partly on maternal antibodies and microbiota colonization for calibrating inflammatory responses (73, 74). As a child grows, repeated antigenic exposures expand and refine B and T cell repertoires, while regulatory checkpoints become more adept at suppressing harmful autoreactivity. In adulthood, most individuals operate under a comparatively stable state of immunological discrimination, although latent interactions with commensal microbes continue to influence tolerance thresholds (75). Over time, however, immunosenescence ensues, particularly evident after mid-life, as thymic involution restricts the output of naive T cells, and cumulative inflammatory signals, sometimes referred to as “inflammaging”, begin to erode the boundary that once reliably separated self from nonself. In advanced age, weaker pathogen defenses coexist with a paradoxical rise in autoreactive phenomena, reflecting an overall decline in regulatory stringency (76, 77). Thus, from fetal tolerance molded by maternal signals to the compromised homeostasis of old age, self and non-self-discriminations evolve as a dynamic negotiation across the human lifespan, continually reshaped by developmental stages, environmental exposures, and intrinsic immunoregulatory capacity. However, over time, the immune system’s capacity to discriminate self from non-self grows increasingly strained, in large part due to a gradual reshaping of lymphocyte populations and a persistent, low-level pro-inflammatory state sometimes called “inflammaging” (78). As the thymus undergoes involution, the output of naive T cells diminishes, leaving an immune system dominated by memory clones that have been repeatedly expanded through earlier antigen exposures. This repertoire skew reduces flexibility and can allow latent autoreactive subsets to proliferate unchecked, especially when regulatory T cells or other immunosuppressive circuits become dysregulated (79). Concurrently, chronic inflammatory signals may expose or modify host antigens, facilitating neo-epitope formation that can trigger novel self-reactivities. Such processes help explain why older adults may face both increased susceptibility to infections, due to diminished adaptive diversity, and a higher incidence of autoimmune phenomena, reflecting the erosion of tolerogenic mechanisms that once maintained a clear boundary between self and nonself. Epigenetic alterations further compound these effects, subtly reprogramming immune cells so that, late in life, the delicate equilibrium between vigilant host defense and self-tolerance is more likely to tip toward either immune hypo-responsiveness or autoreactivity.

In the clinical arena, cancer immunotherapy provided yet another test of the self–non-self-dogma. Tumors arise from host cells, yet subtle genetic or epigenetic changes can make them “foreign enough” to be recognized, or at least recognized once immune checkpoints are lifted (80). The success of checkpoint inhibitors such as anti-PD-1 or anti-CTLA-4 also showed that tolerance mechanisms might be manipulated to break self-tolerance, leading to potent antitumor immunity at the cost of auto-inflammatory side effects. (81) This demonstrates that the boundary between self and altered self is not absolute but can be recalibrated through interventions that modulate immune regulatory pathways. Finally, this concept of self and non-self cannot be exclusively watch from the immune response itself. In fact, growing recognition that immune cells can express neuroendocrine elements, while neurons and glial cells exhibit immune-like functions, has profoundly influenced discussions on how the body senses its internal and external environments, blurring classical notions of self and non-self (49, 82–86). In fact, T lymphocytes and macrophages often bear receptors for neurotransmitters and neuropeptides, enabling them to receive signals traditionally attributed to the nervous system as well as enzymes to convert them and receptors (49, 87–91). Concurrently, microglia and astrocytes in the central nervous system secrete cytokines in ways reminiscent of peripheral immune cells or produce aberrant immunoglobulins (86, 92–95). This mutual expression of molecules from both domains highlights a shared “cognitive” capacity: the nervous and immune systems each interpret a vast array of molecular signals, be they hormonal, neurotransmitter, or pathogen-associated cues and generate responses that maintain organismal homeostasis. In effect, the immune system becomes akin to a sensory organ, attuned not solely to microbial invaders but also to internal physiological shifts, while the nervous system extends beyond classical neurotransmission to engage in immunomodulatory functions. Such cross-talk challenges any simple self–non-self-boundary by revealing that “self” is constantly redefined through neuroendocrine-immune circuits that monitor, interpret, and respond to signals across multiple physiological layers (96). An illustration of this intertwining could depict neurons and T cells exchanging molecular signals across a blood–brain barrier interface, symbolizing how immune and nervous systems integration transforms a rigid model of discrimination into a more flexible, context-dependent negotiation of identity (97).

Thus, the accumulation of all these findings places the field at a crossroads. While the concept of self and non-self remains ingrained in immunological discourse, it is increasingly clear that its classical forms do not fully capture the intricacies of immune function. Researchers who were once taught a dogmatic approach are now discussing the immune system as a dynamic, context-sensitive network that modulates its responses in response to signals from stress, context, microbial symbiosis, epigenetic memory, and local immunoregulatory conditions. The question is no longer whether classical dogma explains all of immunology, which it clearly does not, but rather how we can integrate its useful elements into a broader framework that recognizes the complexity of reality.

## Toward an integrative vision and the question of the dogma’s demise

The final and perhaps most pressing issue is whether the self-non-self-dogma has effectively reached its end, or whether it can persist in some modified form. To answer this question, one must consider how contemporary immunological phenomena converge to undermine or refine the dogma, and what theoretical constructs have emerged in its place. The dynamic equilibrium between immune tolerance and aggression continuously adjusts according to microbial exposure, metabolic cues, developmental signals, and tissue integrity indicators (61, 98–101). Evidence from trained immunity research (51) underscores innate immunity’s dynamic interactions with adaptive immunity, involving complex epigenetic and metabolic pathways (102), significantly reshaping immune memory and responsiveness. There are several arguments for the continued relevance of self/non-self-discrimination in a cautious, updated guise. First, in clinical contexts, particularly in transplantation and autoimmunity, the notion that a transplanted organ or autoreactive immune repertoire is “foreign enough” to elicit an immune response retains descriptive power (103). In practice, the manipulation of T cell reactivity or the design of immunosuppressive regimens is often guided by the principle that autoreactivity must be suppressed and recognition of foreign tissue must be minimized. This practical utility suggests that the dogma, while incomplete, remains a useful heuristic. Second, the structure of immune receptors, both adaptive and innate, suggests a system designed to recognize molecular shapes not typically found in the host. Pattern recognition receptors target microbial motifs, T and B cell receptors are selected to avoid strong self-reactivity, and MHC restriction still sets important limits (102). These observations lend credibility to the idea that an underlying discrimination mechanism remains central, even if it has been partially overshadowed by more dynamic layers of interpretation. In other words, self-non-self-discrimination may not be the whole story, but it is not entirely wrong. However, the strong case for a paradigmatic shift cannot be ignored. Conceptually, the entire premise that the immune system’s first job is to define what is self so as not to attack it is contradicted when we consider microchimerism and pregnancy tolerance, cases in which truly foreign (paternally derived) tissues persist without triggering rejection (104). Similarly, the fact that the human microbiome can be vital to normal function challenges the assumption that foreignness inevitably triggers aggression. Instead, more recent frameworks suggest that the role of the immune system is to manage complex ecological relationships by distinguishing beneficial or neutral foreign entities from harmful ones. The presence of “harmless foreign” elements is a mainstay in the gut, skin, and oropharynx. Moreover, the integration of viruses into the genome, sometimes with evolutionary and developmental benefits, blurs the boundary between self and foreign in a fundamental, genomic sense. Endogenous retroviral elements constitute a significant portion of human DNA, yet no robust immune aggression is mounted against these deeply embedded viral sequences (105). This phenomenon invites researchers to conceive of “self” as including certain categories of foreign genetic material that have become symbiotic or neutral over evolutionary time. These concepts are consistent with the contemporary philosophy of immunology, which incorporates ecological and developmental insights, such as the observation that commensal microbes, fetal cells in the maternal circulation, or latent viruses are not automatically rejected as “non-self,” but instead coexist with the host under specific regulatory conditions. This move away from a rigid self-non-self-opposition repositions immunity as a process of constant negotiation between entities and signals, one in which boundaries are actively policed, redefined, or relaxed depending on the local context (61, 100, 105). Some philosophers and biologists extend the conversation further by considering “holobiont” or “multispecies” identity, where the host is viewed as a consortium of its own cells plus its microbial partners (106–109). In this view, immune mechanisms do not simply defend an isolated organism; they manage an ecological system that we have conventionally referred to as the “body”. In other words, the activation thresholds and responses of the immune system are strongly shaped by local danger signals, tissue stress, symbiotic cues, or immunomodulatory molecules. This is without an absolute distinction between endogenous and exogenous molecules. The boundaries are drawn and redrawn by ongoing interactions, life events, and environmental exposures. Such frameworks have implications for how we interpret immune pathologies and therapies. They suggest, for example, that autoimmunity may reflect not just a “mistake” in identification, such as in hygienist theory but a breakdown in regulatory negotiations; and that the success of transplantation or maternal-fetal tolerance depends on the establishment of immunological relationships, not just the suppression of foreign recognition. By conceptualizing the immune system as an evolving, context-responsive web of interactions, these new philosophical discourses invite us to expand our understanding of identity, no longer viewing “self” as an inviolable domain, but as a dynamic process shaped by biological, environmental, and sociocultural factors. Furthermore, the application of systems immunology has uncovered complex gene expression networks and feedback loops that cannot be easily reduced to a binary logic of self/non-self. High-dimensional single-cell analyses reveal that immune cells pass through multiple functional states, often influenced by metabolic signals, epigenetic marks, and microbial crosstalk. The fluidity of these states defies any simple classification of which antigens are self or non-self. Even the earliest proponents of the dogma could not have predicted the depth of complexity that modern computational and omics approaches would reveal. So, is the self/non-self-dogma finished? From one perspective, one could argue that it is indeed outdated as a universal explanatory framework. Too many exceptions, microchimerism, innate immunity, danger signals, commensals, hygienist theory, prove that discrimination is more about context than absolute strangeness. Indeed, many immunologists now refer to the dogma as a historical steppingstone, a once-powerful lens that must be replaced by more nuanced theories. From another perspective, however, it may be more accurate to say that the dogma has simply been reinterpreted and incorporated into broader paradigms. The notion that the immune system must not attack healthy tissues and must eliminate harmful invaders remains clinically and biologically relevant. However, the criteria for deciding what is self and what is not are determined solely by clonally encoded specificity or predefined sets of self-antigens. Instead, the immune system uses a repertoire of signals - metabolic, microbial, epigenetic - to dynamically calibrate responses.

Thus, the current debate is not so much whether self-non-self-discrimination is right or wrong, but whether it is sufficient to capture immunological reality. The consensus seems to be that it is not, and that we need a richer framework that incorporates microbial ecology, tissue-specific environments, damage sensing, and the capacity for immune re-education. If there is an “end of a dogma,” it lies in the realization that self/non-self is not the sole or even primary axis around which immune phenomena revolve. Instead, we have arrived at a place where an integrative, contextual, and ecologically aware immunology can better explain phenomena that were once puzzling exceptions. Considering these considerations, the present moment is one of revision and expansion, rather than wholesale rejection, of the classical conceptual framework. Self-non-self remains a useful reference point, especially for teaching and certain medical applications, but it no longer has an exclusive claim to immunological truth. Over the past seventy years, the field has moved from a dogmatic stance to an expanded appreciation of complexity and nuance. Finally, when the immune and nervous systems are viewed as sharing a “cognitive” capacity, the immune system becomes akin to a sensory organ, attuned not only to microbial invaders but also to internal physiological changes, while the nervous system extends beyond classical neurotransmission to engage in immunomodulatory functions. Such crosstalk challenges any simple self-non-self-boundary by revealing that “self” is constantly redefined by neuroendocrine-immune circuits that monitor, interpret, and respond to signals across multiple physiological layers. The next decades are likely to witness further integration of multi-omics data, advanced computational modeling, and ecological principles, solidifying an immunologically integrated view in which the “end of dogma” does not mean a collapse into chaos, but a maturing discipline flexible enough to accommodate real-world complexities.

## Conclusion

**Figure 1:**
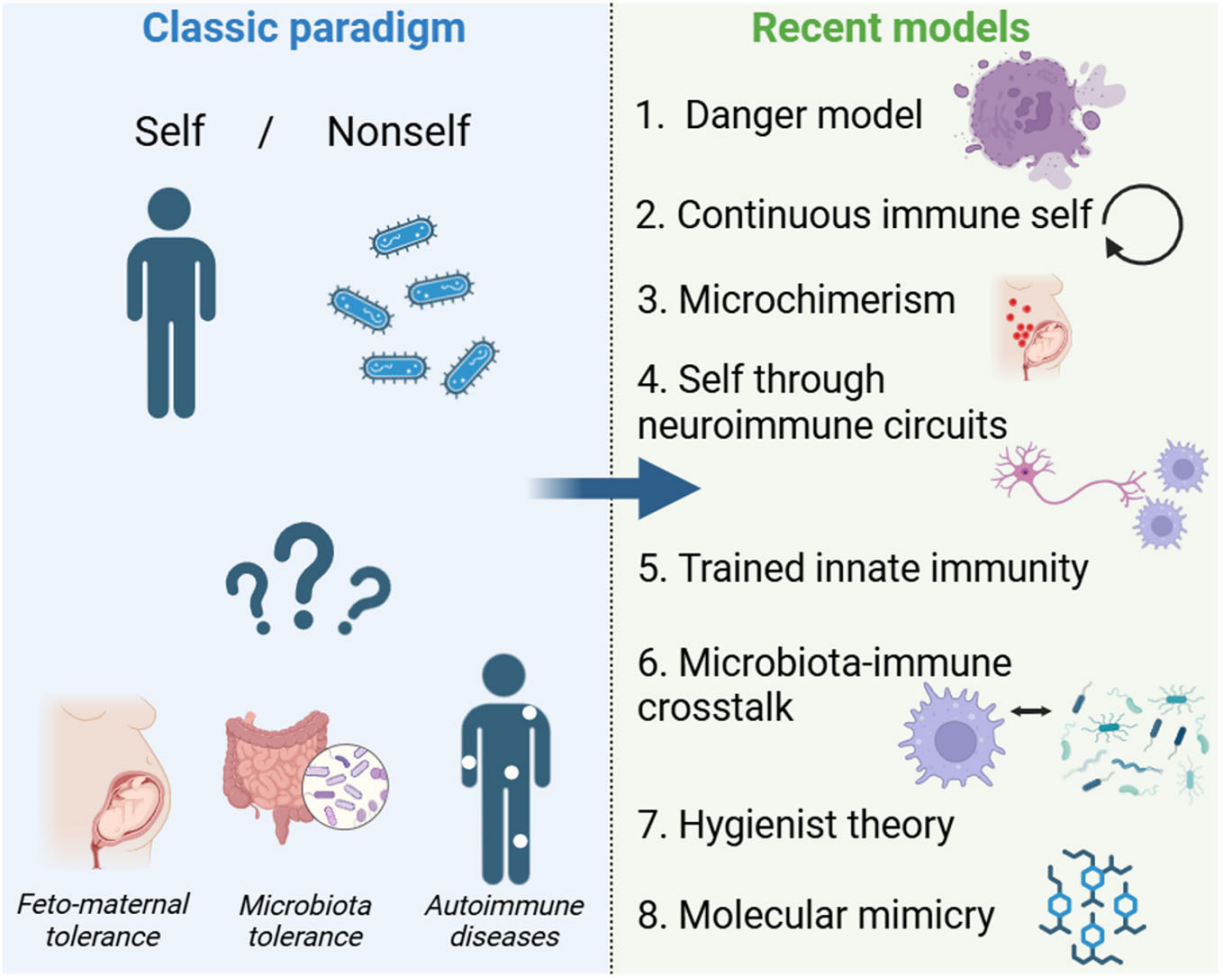
From Self/Nonself to Modern Immune Models. This figure compares the classic self/nonself paradigm with recent immunological models. The traditional view, where the immune system distinguishes between “self” and “nonself,” struggles to explain feto-maternal tolerance, microbiota tolerance, and autoimmunity. Newer models have emerged, including the danger model, continuous immune self, microchimerism, neuroimmune circuits, trained innate immunity, and microbiota-immune crosstalk. The arrow represents the conceptual shift from a binary framework to a more dynamic understanding of immunity.

The evolution of the self/non-self-paradigm in immunology provides a fascinating case study of how scientific dogmas emerge, solidify, and then either adapt or fade under the weight of new evidence. From early philosophical questions about personal identity, through the microbiological revolutions of Pasteur and Koch, to Burnet’s theory of clonal selection and subsequent breakthroughs in transplantation, the notion that the central purpose of the immune system is to recognize and eradicate the foreign while preserving the self has served as a unifying principle. But in the late twentieth century, challenges began to emerge. The recognition that innate immunity is not merely non-specific but is guided by pattern recognition and danger signals; the demonstration that pregnancy, commensal microbes, hygienist theory and microchimerism complicate the boundary between self and non-self; and the discovery that the immune system itself can be epigenetically reprogrammed have all forced major theoretical adjustments. Today, few would deny the usefulness of the original concept in certain contexts, but even fewer would proclaim it a complete account of immune regulation. Instead, we now view the immune system as an adaptive network that continuously negotiates what is beneficial, harmful, or neutral in concert with various tissues, microbial partners, and environmental cues. This dynamic perspective does not completely dismantle the notion of self/non-self but rather incorporates it into a richer tapestry of signals and regulatory mechanisms. Whether this represents the “end of a dogma” may depend on one’s definitions. For those who cling to a strict, binary interpretation, it is indeed the end. For those who see dogmas as evolving scientific constructs, the journey of self-non-self-discrimination has simply branched off into more nuanced terrain. Ultimately, the enduring legacy of the self-nonself model is that it provided a powerful organizing framework that has been indispensable in shaping immunological research for decades. Its partial obsolescence is, in fact, a tribute to how far immunology has come. As we move into deeper explorations of microbiome-immune interactions and epigenetic plasticity, the field will undoubtedly continue to change. The fundamental question of how an organism maintains its integrity in an ever-changing environment of microbes, tissues, and signals remains as relevant as ever, but the answers we seek must match the complexity and dynamism of biological reality. If this means embracing the “end of a dogma,” it also heralds the dawn of a more integrative immunological science (**Figure 1**).
